# 2-(4-Hy­droxy­phen­yl)-1*H*-benzimidazol-3-ium chloride monohydrate

**DOI:** 10.1107/S1600536813023441

**Published:** 2013-08-31

**Authors:** Jazmin E. González-Padilla, Martha Cecila Rosales-Hernández, Itzia I. Padilla-Martínez, Efren V. García-Báez, Susana Rojas-Lima

**Affiliations:** a Laboratorio de Biofisica y Biocatálisis, Sección de Estudios de Posgrado e Investigación de la Escuela Superior de Medicina del Instituto Politécnico Nacional, Plan de San Luis y Díaz Mirón s/n Casco de Santo Tomás, México, DF 11340, Mexico; b Laboratorio de Investigación en Química, Departamento de Ciencias Básicas, Unidad Profesional Interdisciplinaria de Biotecnología del Instituto Politécnico Nacional, Av. Acueducto s/n Barrio la laguna Ticoman, México, DF 07340, Mexico; c Centro de Investigaciones Químicas, Universidad Autonoma del Estado de Hidalgo, km. 4.5 Carretera Pachuca-Tulancingo, Mineral de la Reforma, Hidalgo 42184, Mexico

## Abstract

The title mol­ecular salt, C_13_H_11_N_2_O^+^·Cl^−^·H_2_O, crystallizes as a monohydrate. In the cation, the phenol and benzimidazole rings are almost coplanar, making a dihedral angle of 3.18 (4)°. The chloride anion and benzimidazole cation are linked by two N^+^—H⋯Cl^−^ hydrogen bonds, forming chains propagating along [010]. These chains are linked through O—H⋯Cl hydrogen bonds involving the water mol­ecule and the chloride anion, which form a diamond core, giving rise to the formation of two-dimensional networks lying parallel to (10-2). Two π–π inter­actions involving the imidazolium ring with the benzene and phenol rings [centroid–centroid distances = 3.859 (3) and 3.602 (3) Å, respectively], contribute to this second dimension. A strong O—H⋯O hydrogen bond involving the water mol­ecule and the phenol substituent on the benzimidazole unit links the networks, forming a three-dimensional structure.

## Related literature

For biological properties of benzimidazoles and their applications, see: Ansari & Lal (2009 ▶); Laryea *et al.* (2010 ▶); Mohan *et al.* (2011 ▶); Refaat (2010 ▶); Zhou *et al.* (2013 ▶); Khan *et al.* (2012 ▶). For their use in crystal-engineering, see: Cai *et al.* (2002 ▶). For standard bond lengths, see: Allen *et al.* (1987 ▶). For the structures of benzimidazole halohydrates, see: Akkurt *et al.* (2010 ▶); Baktır *et al.* (2010 ▶). For the microwave synthesis of neutral 4-(1*H*-benzimidazol-2-yl)phenol, see: Navarrete-Vázquez *et al.* (2006 ▶). For its crystal structure, see: Zhan *et al.* (2007 ▶).
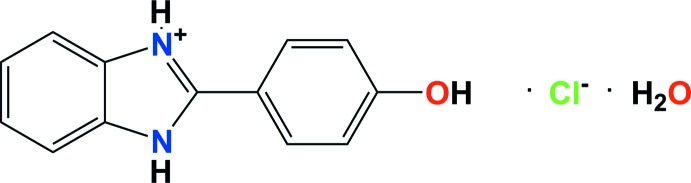


## Experimental

### 

#### Crystal data

C_13_H_11_N_2_O^+^·Cl^−^·H_2_O
*M* *_r_* = 264.70
Monoclinic, 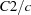

*a* = 10.3225 (5) Å
*b* = 16.3159 (5) Å
*c* = 15.4618 (8) Å
β = 101.071 (5)°
*V* = 2555.6 (2) Å^3^
*Z* = 8
Mo *K*α radiation
μ = 0.29 mm^−1^
*T* = 293 K
0.38 × 0.33 × 0.28 × 0.15 (radius) mm

#### Data collection

Oxford Diffraction Xcalibur Ruby Gemini diffractometer
Absorption correction: for a sphere [the interpolation procedure of (Dwiggins, 1975 ▶) was used with some modification] *T*_min_ = 0.861, *T*_max_ = 0.862
12720 measured reflections
2519 independent reflections
2032 reflections with *I* > 2σ(*I*)
*R*_int_ = 0.028

#### Refinement

*R*[*F* ^2^ > 2σ(*F* ^2^)] = 0.043
*wR*(*F* ^2^) = 0.124
*S* = 1.06
2519 reflections
163 parameters
H-atom parameters constrained
Δρ_max_ = 0.24 e Å^−3^
Δρ_min_ = −0.19 e Å^−3^

### 

Data collection: *CrysAlis CCD* (Oxford Diffraction, 2009 ▶); cell refinement: *CrysAlis CCD*; data reduction: *CrysAlis RED* (Oxford Diffraction, 2009 ▶); program(s) used to solve structure: *SHELXS97* (Sheldrick, 2008 ▶); program(s) used to refine structure: *SHELXL97* (Sheldrick, 2008 ▶); molecular graphics: *Mercury* (Macrae *et al.*, 2006 ▶); software used to prepare material for publication: *SHELXL97*, *WinGX* (Farrugia, 2012 ▶) and *publCIF* (Westrip, 2010 ▶).

## Supplementary Material

Crystal structure: contains datablock(s) I, New_Global_Publ_Block. DOI: 10.1107/S1600536813023441/su2637sup1.cif

Structure factors: contains datablock(s) I. DOI: 10.1107/S1600536813023441/su2637Isup2.hkl

Supplementary material file. DOI: 10.1107/S1600536813023441/su2637Isup3.cml

Additional supplementary materials:  crystallographic information; 3D view; checkCIF report

## Figures and Tables

**Table 1:** Hydrogen-bond geometry (Å, °)

*D*—H⋯*A*	*D*—H	H⋯*A*	*D*⋯*A*	*D*—H⋯*A*
N1—H1⋯Cl1	0.86	2.29	3.1167 (16)	162
N3—H3⋯Cl1^i^	0.86	2.32	3.1625 (16)	168
O1—H1*A*⋯Cl1^ii^	0.89	2.39	3.266 (2)	167
O1—H1*B*⋯Cl1	0.92	2.33	3.243 (2)	171
O13—H13⋯O1^iii^	0.82	1.86	2.666 (3)	166

Symmetry codes: (i) 
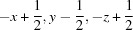
; (ii) 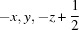; (iii) 
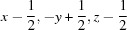
.

**Fig. 1.**
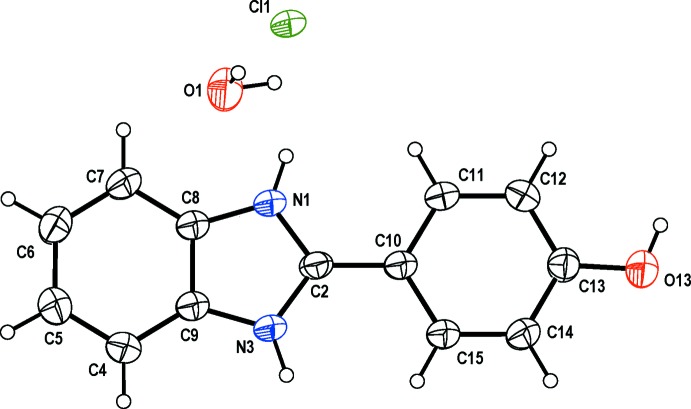
The molecular structure of the title compound, with atom labelling. The displacement ellipsoids are drawn at the 30% probability level.

**Fig. 2.**
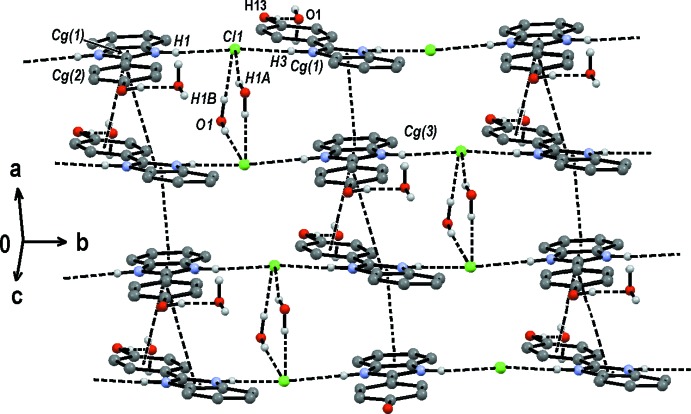
The crystal packing of the title compound viewed along direction [101]. The hydrogen bonds and centroid-centroid interactions are shown as dashed lines (see Table 1 for details). H atoms not involved in hydrogen bonding have been omitted for clarity.

## supplementary crystallographic information

### 1. Comment

Benzimidazoles are a class of compounds with a wide variety of biological properties (Mohan *et al.*, 2011; Refaat, 2010; Laryea *et al.*, 2010; Ansari & Lal, 2009) and have applications in crystal-engineering (Cai *et al.*, 2002). Particularly, the neutral derivative of the title compound has recently been reported as having good antimicrobial (Zhou *et al.*, 2013) and β-glucuronidase inhibitory activity (Khan *et al.*, 2012). Herein we report on the crystal structure of the title compound.

The title compound crystallizes as the monohydrate of a hydrochloride salt, Fig. 1. Bond lengths (Allen *et al.*, 1987) and angles are within normal ranges. The phenol and benzimidazole rings are almost coplanar with a dihedral angle of 3.18 (4) °.

One water molecule was included in the asymmetric unit which, besides the chloride anion, directs the organization in the lattice forming hydrogen bonding interactions (Table 1 and Fig. 2), as has been observed in other halohydrates (Akkurt *et al.*, 2010; Baktır *et al.*, 2010).

In the crystal, the chloride anion and the benzimidazole molecule give rise to the first dimension through two N^+^—H···Cl^-^ interactions (Table 1); forming chains propagating along [010].

The water molecule and chloride anion form a diamond core through O—H···Cl hydrogen bonds, giving rise to the second dimension (Table 1); forming two-dimensional networks lying parallel to plane (10-2).

Two contributions from aromatic systems of the type π–π between the electronic deficient imidazolium ring, [N1/N2/C2/C8/C9 with centroid Cg1], with both electronic rich benzene, [C4-C9 with centroid Cg2], and phenol, [C10-C15 with centroid Cg3], rings contribute to the development in the second dimension [Cg1···Cg2^i^ = 3.6017 (13) Å and Cg1···Cg3^ii^ = 3.8593 (12) Å; symmetry codes: (i) -x+1, y, -z+1/2; (ii) -x, y, -z+1/2].

The third dimension is built by a strong O-H···O hydrogen bond between the water molecule, as the acceptor, with the phenol group of benzimidazole, as donor (Table 1 and Fig. 2); forming a three-dimensional structure.

The molecular structure of the title compound is similar to that of the neutral compound 4-(1*H*-benzimidazol-2-yl)phenol (Zhan *et al.*, 2007), where the dihedral angle between the benzimidazole ring system and the phenol ring is 8.11 (5) °. In the crystal lattice, only N—H···O and O—H···N (benzimidazole-phenol) hydrogen bonds are present.

### 2. Experimental

The neutral derivative of the title compound, 4-(1*H*-benzimidazol-2-yl)phenol, was synthesized following a reported procedure (Navarrete-Vázquez *et al.*, 2006). The reaction of 0.257 g (2.38 mmol) of 1,3-phenylenediamine, 0.290 g (2.38 mmol) of 4-hydroxybenzaldehyde and 0.452 g (2.38 mmol) of Na_2_S_2_O_5_ in 4 ml of DMSO as solvent, heated at 423 K for 15 min in a microwave oven gave a 94% yield of the neutral compound. Colourless block-like crystals of the title compound were obtained by crystallization of this neutral compound in a THF solution with a few drops of an aqueous solution of HCl (10%).

### 3. Refinement

The OH, water and NH H atoms could be located in Fourier difference maps. The water H atoms were refined as riding atoms with *U*_iso_(H)= 1.5U_eq_(O). In the final cycles of refinement the OH, NH and C-bound H atoms were positioned geometrically and treated as riding atoms: O-H = 0.82 Å, N—H = 0.86 Å, C—H = 0.93 Å for CH H atoms, with *U*_iso_(H) = 1.5U_eq_(O) and = 1.2U_eq_(N,C) for other H atoms.

### Crystal data

**Table:**

C_13_H_11_N_2_O^+^·Cl^−^·H_2_O	*F*(000) = 1104
*M**_r_* = 264.70	*D*_x_ = 1.376 Mg m^−^^3^
Monoclinic, *C*2/*c*	Mo *K*α radiation, λ = 0.71073 Å
Hall symbol: -C 2yc	Cell parameters from 600 reflections
*a* = 10.3225 (5) Å	θ = 20–25°
*b* = 16.3159 (5) Å	µ = 0.29 mm^−^^1^
*c* = 15.4618 (8) Å	*T* = 293 K
β = 101.071 (5)°	Block, colourless
*V* = 2555.6 (2) Å^3^	0.38 × 0.33 × 0.28 × 0.15 (radius) mm
*Z* = 8	

### Data collection

**Table:**

Oxford Diffraction Xcalibur Ruby Gemini diffractometer	2519 independent reflections
Graphite monochromator	2032 reflections with *I* > 2σ(*I*)
Detector resolution: 10.434 pixels mm^-1^	*R*_int_ = 0.028
ω scans	θ_max_ = 26.2°, θ_min_ = 2.4°
Absorption correction: for a sphere [the interpolation procedure of (Dwiggins, 1975) was used with some modification]	*h* = −12→10
*T*_min_ = 0.861, *T*_max_ = 0.862	*k* = −20→20
12720 measured reflections	*l* = −19→19

### Refinement

**Table:**

Refinement on *F*^2^	Primary atom site location: structure-invariant direct methods
Least-squares matrix: full	Secondary atom site location: difference Fourier map
*R*[*F*^2^ > 2σ(*F*^2^)] = 0.043	Hydrogen site location: inferred from neighbouring sites
*wR*(*F*^2^) = 0.124	H-atom parameters constrained
*S* = 1.06	*w* = 1/[σ^2^(*F*_o_^2^) + (0.0595*P*)^2^ + 1.2001*P*] where *P* = (*F*_o_^2^ + 2*F*_c_^2^)/3
2519 reflections	(Δ/σ)_max_ = 0.002
163 parameters	Δρ_max_ = 0.24 e Å^−^^3^
0 restraints	Δρ_min_ = −0.19 e Å^−^^3^

### Special details

**Table:**

Geometry. Bond distances, angles *etc*. have been calculated using the rounded fractional coordinates. All su's are estimated from the variances of the (full) variance-covariance matrix. The cell e.s.d.'s are taken into account in the estimation of distances, angles and torsion angles
Refinement. Refinement of *F*^2^ against ALL reflections. The weighted *R*-factor *wR* and goodness of fit *S* are based on *F*^2^, conventional *R*-factors *R* are based on *F*, with *F* set to zero for negative *F*^2^. The threshold expression of *F*^2^ > σ(*F*^2^) is used only for calculating *R*-factors(gt) *etc*. and is not relevant to the choice of reflections for refinement. *R*-factors based on *F*^2^ are statistically about twice as large as those based on *F*, and *R*- factors based on ALL data will be even larger.

### Fractional atomic coordinates and isotropic or equivalent isotropic displacement parameters (Å^2^)

**Table:**

	*x*	*y*	*z*	*U*_iso_*/*U*_eq_	
O13	−0.24260 (17)	0.03354 (10)	−0.00034 (11)	0.0774 (6)	
N1	0.26708 (17)	0.19303 (9)	0.25356 (11)	0.0574 (5)	
N3	0.30346 (16)	0.06482 (9)	0.28407 (11)	0.0555 (5)	
C2	0.2213 (2)	0.11736 (10)	0.23396 (13)	0.0513 (6)	
C4	0.5115 (2)	0.08213 (13)	0.39988 (15)	0.0675 (8)	
C5	0.5938 (3)	0.14242 (15)	0.44198 (17)	0.0740 (9)	
C6	0.5692 (3)	0.22515 (15)	0.42173 (17)	0.0773 (9)	
C7	0.4630 (3)	0.25048 (13)	0.35978 (17)	0.0724 (8)	
C8	0.3799 (2)	0.19009 (11)	0.31768 (13)	0.0565 (6)	
C9	0.4039 (2)	0.10732 (11)	0.33766 (13)	0.0559 (6)	
C10	0.1031 (2)	0.09650 (11)	0.17127 (12)	0.0522 (6)	
C11	0.0230 (2)	0.15718 (12)	0.12455 (15)	0.0647 (7)	
C12	−0.0923 (2)	0.13790 (13)	0.06684 (15)	0.0661 (8)	
C13	−0.1307 (2)	0.05700 (13)	0.05431 (13)	0.0587 (7)	
C14	−0.0528 (2)	−0.00400 (12)	0.10052 (15)	0.0643 (7)	
C15	0.0623 (2)	0.01506 (11)	0.15739 (14)	0.0585 (7)	
O1	0.12352 (19)	0.35642 (11)	0.39008 (13)	0.0917 (7)	
Cl1	0.19078 (6)	0.37204 (3)	0.19429 (4)	0.0720 (2)	
H1	0.23133	0.23718	0.22961	0.0688*	
H3	0.29490	0.01237	0.28299	0.0665*	
H4	0.52758	0.02694	0.41271	0.0809*	
H5	0.66666	0.12785	0.48448	0.0888*	
H6	0.62665	0.26436	0.45120	0.0927*	
H7	0.44771	0.30569	0.34671	0.0868*	
H11	0.04805	0.21183	0.13257	0.0776*	
H12	−0.14403	0.17927	0.03646	0.0793*	
H13	−0.27318	0.07238	−0.03130	0.1160*	
H14	−0.07904	−0.05847	0.09280	0.0771*	
H15	0.11382	−0.02671	0.18711	0.0702*	
H1A	0.03995	0.35369	0.36137	0.1376*	
H1B	0.15177	0.35970	0.33730	0.1376*	

### Atomic displacement parameters (Å^2^)

**Table:**

	*U*^11^	*U*^22^	*U*^33^	*U*^12^	*U*^13^	*U*^23^
O13	0.0655 (11)	0.0665 (10)	0.0901 (11)	−0.0031 (7)	−0.0101 (8)	−0.0040 (8)
N1	0.0608 (11)	0.0332 (7)	0.0725 (10)	0.0002 (6)	−0.0011 (8)	0.0030 (6)
N3	0.0593 (11)	0.0332 (7)	0.0704 (10)	−0.0001 (6)	0.0036 (8)	0.0027 (6)
C2	0.0570 (12)	0.0358 (8)	0.0613 (10)	−0.0001 (7)	0.0116 (9)	0.0013 (7)
C4	0.0654 (15)	0.0513 (11)	0.0802 (14)	0.0045 (9)	0.0003 (11)	0.0078 (10)
C5	0.0639 (16)	0.0714 (14)	0.0795 (15)	0.0028 (11)	−0.0043 (12)	0.0006 (11)
C6	0.0703 (17)	0.0622 (13)	0.0914 (16)	−0.0098 (11)	−0.0043 (12)	−0.0126 (11)
C7	0.0752 (16)	0.0419 (10)	0.0929 (15)	−0.0047 (9)	−0.0016 (12)	−0.0066 (10)
C8	0.0580 (13)	0.0413 (9)	0.0676 (11)	−0.0001 (8)	0.0053 (9)	0.0012 (8)
C9	0.0573 (13)	0.0423 (9)	0.0662 (11)	−0.0003 (8)	0.0072 (9)	0.0020 (8)
C10	0.0565 (12)	0.0399 (9)	0.0596 (10)	0.0007 (8)	0.0097 (9)	0.0016 (7)
C11	0.0710 (15)	0.0389 (9)	0.0784 (13)	−0.0029 (9)	−0.0002 (11)	0.0037 (9)
C12	0.0643 (15)	0.0545 (11)	0.0738 (13)	0.0066 (9)	−0.0010 (11)	0.0102 (9)
C13	0.0531 (13)	0.0574 (11)	0.0633 (11)	−0.0001 (9)	0.0056 (9)	−0.0040 (8)
C14	0.0651 (14)	0.0437 (10)	0.0799 (13)	−0.0025 (9)	0.0034 (11)	−0.0049 (9)
C15	0.0589 (13)	0.0410 (9)	0.0719 (12)	0.0026 (8)	0.0030 (10)	0.0009 (8)
O1	0.0786 (13)	0.0885 (12)	0.0977 (13)	−0.0048 (9)	−0.0090 (10)	−0.0099 (10)
Cl1	0.0792 (4)	0.0328 (3)	0.0941 (4)	−0.0001 (2)	−0.0079 (3)	0.0024 (2)

### Geometric parameters (Å, º)

**Table:**

O13—C13	1.349 (3)	C8—C9	1.397 (3)
O13—H13	0.8200	C10—C11	1.399 (3)
O1—H1B	0.9200	C10—C15	1.398 (3)
O1—H1A	0.8900	C11—C12	1.380 (3)
N1—C8	1.378 (3)	C12—C13	1.381 (3)
N1—C2	1.336 (2)	C13—C14	1.388 (3)
N3—C9	1.383 (3)	C14—C15	1.371 (3)
N3—C2	1.342 (2)	C4—H4	0.9300
N1—H1	0.8600	C5—H5	0.9300
N3—H3	0.8600	C6—H6	0.9300
C2—C10	1.446 (3)	C7—H7	0.9300
C4—C5	1.379 (3)	C11—H11	0.9300
C4—C9	1.385 (3)	C12—H12	0.9300
C5—C6	1.398 (3)	C14—H14	0.9300
C6—C7	1.374 (4)	C15—H15	0.9300
C7—C8	1.384 (3)		
			
C13—O13—H13	109.00	C10—C11—C12	121.57 (18)
H1A—O1—H1B	90.00	C11—C12—C13	119.82 (19)
C2—N1—C8	110.15 (16)	C12—C13—C14	119.39 (19)
C2—N3—C9	110.09 (15)	O13—C13—C12	123.18 (19)
C2—N1—H1	125.00	O13—C13—C14	117.43 (19)
C8—N1—H1	125.00	C13—C14—C15	120.82 (18)
C2—N3—H3	125.00	C10—C15—C14	120.82 (18)
C9—N3—H3	125.00	C5—C4—H4	121.00
N1—C2—N3	107.61 (17)	C9—C4—H4	121.00
N1—C2—C10	125.87 (17)	C6—C5—H5	120.00
N3—C2—C10	126.51 (16)	C4—C5—H5	120.00
C5—C4—C9	117.1 (2)	C5—C6—H6	119.00
C4—C5—C6	121.0 (3)	C7—C6—H6	119.00
C5—C6—C7	122.2 (2)	C8—C7—H7	122.00
C6—C7—C8	117.0 (2)	C6—C7—H7	121.00
N1—C8—C7	132.49 (18)	C10—C11—H11	119.00
C7—C8—C9	121.1 (2)	C12—C11—H11	119.00
N1—C8—C9	106.40 (16)	C13—C12—H12	120.00
N3—C9—C4	132.57 (17)	C11—C12—H12	120.00
C4—C9—C8	121.68 (18)	C13—C14—H14	120.00
N3—C9—C8	105.76 (17)	C15—C14—H14	120.00
C11—C10—C15	117.56 (18)	C10—C15—H15	120.00
C2—C10—C15	121.15 (17)	C14—C15—H15	120.00
C2—C10—C11	121.25 (17)		
			
C8—N1—C2—N3	−0.5 (2)	C6—C7—C8—N1	−179.9 (2)
C8—N1—C2—C10	178.44 (19)	C6—C7—C8—C9	−0.1 (4)
C2—N1—C8—C7	−179.9 (3)	N1—C8—C9—N3	0.0 (2)
C2—N1—C8—C9	0.3 (2)	N1—C8—C9—C4	179.59 (19)
C9—N3—C2—N1	0.5 (2)	C7—C8—C9—N3	−179.8 (2)
C9—N3—C2—C10	−178.43 (19)	C7—C8—C9—C4	−0.2 (3)
C2—N3—C9—C4	−179.8 (2)	C2—C10—C11—C12	−177.8 (2)
C2—N3—C9—C8	−0.3 (2)	C15—C10—C11—C12	0.1 (3)
N1—C2—C10—C11	−0.6 (3)	C2—C10—C15—C14	177.3 (2)
N1—C2—C10—C15	−178.4 (2)	C11—C10—C15—C14	−0.6 (3)
N3—C2—C10—C11	178.1 (2)	C10—C11—C12—C13	0.0 (3)
N3—C2—C10—C15	0.3 (3)	C11—C12—C13—O13	179.4 (2)
C9—C4—C5—C6	−0.4 (4)	C11—C12—C13—C14	0.4 (3)
C5—C4—C9—N3	180.0 (2)	O13—C13—C14—C15	−179.9 (2)
C5—C4—C9—C8	0.5 (3)	C12—C13—C14—C15	−0.9 (3)
C4—C5—C6—C7	0.1 (4)	C13—C14—C15—C10	1.0 (3)
C5—C6—C7—C8	0.1 (4)		

### Hydrogen-bond geometry (Å, º)

**Table:**

*D*—H···*A*	*D*—H	H···*A*	*D*···*A*	*D*—H···*A*
N1—H1···Cl1	0.86	2.29	3.1167 (16)	162
N3—H3···Cl1^i^	0.86	2.32	3.1625 (16)	168
O1—H1*A*···Cl1^ii^	0.89	2.39	3.266 (2)	167
O1—H1*B*···Cl1	0.92	2.33	3.243 (2)	171
O13—H13···O1^iii^	0.82	1.86	2.666 (3)	166

Symmetry codes: (i) −*x*+1/2, *y*−1/2, −*z*+1/2; (ii) −*x*, *y*, −*z*+1/2; (iii) *x*−1/2, −*y*+1/2, *z*−1/2.
